# A Rare Case of Cannabis-induced Acute Pancreatitis

**DOI:** 10.7759/cureus.4878

**Published:** 2019-06-11

**Authors:** Sami Ghazaleh, Ali Alqahtani, Christian Nehme, Aya Abugharbyeh, Tamer S Said Ahmed

**Affiliations:** 1 Internal Medicine, University of Toledo Medical Center, Toledo, USA; 2 Pulmonary / Critical Care Medicine, University of Toledo Medical Center, Toledo, USA

**Keywords:** cannabis, acute pancreatitis, marijuana

## Abstract

Acute pancreatitis is a sudden inflammatory condition of the pancreas, caused mainly by gallstones and alcohol abuse. A significant proportion of acute pancreatitis cases remain idiopathic. Recent reports have highlighted cannabis use as an etiology of acute pancreatitis. A few case reports are available that report the association of cannabis with acute pancreatitis. Considering the global use of cannabis medically and illicitly, it becomes imperative to explore this adverse effect of cannabis use especially in idiopathic cases of acute pancreatitis. Here, in this report, we present a case of acute pancreatitis with no obvious cause. The patient was a 48-year-old female with no history of alcohol use. She had a history of cholecystectomy with normal serum triglycerides and calcium levels. The patient was consuming marijuana (cannabis) daily for the last three years. The diagnosis of cannabis-induced acute pancreatitis was made in the patient after other causes were excluded. It is difficult to distinguish cannabis-induced pancreatitis as there are no clear and specific associated clinical features. The diagnosis of cannabis-induced pancreatitis becomes even more challenging due to the use of multiple drugs. It becomes difficult to point out the causative agent among the multitude of drugs. Hence, a detailed history of drug intake in cases of acute pancreatitis may help to identify the candidature of the drugs in the pathogenesis of the disease. In view of the increasing illicit and medical use of cannabis, it becomes quintessential for clinicians to consider pancreatitis as a possible adverse effect of cannabis.

## Introduction

Acute pancreatitis is a common condition that has high morbidity and mortality [1]. An effort is usually made to identify the etiology of acute pancreatitis to guide therapy and prevent a recurrence. In the United States, acute pancreatitis is commonly caused by gallstones, alcohol, hypertriglyceridemia, hypercalcemia, post-endoscopic retrograde cholangiopancreatography (ERCP), medications, and infections [2]. When a cause cannot be identified, the condition is referred to as idiopathic pancreatitis, which constitutes about 15-25% of acute pancreatitis cases [3].

Several cases have been reported in the literature linking cannabis use with the development of acute pancreatitis [4-6]. In the literature, cannabis-induced acute pancreatitis was diagnosed in patients with a history of marijuana use who underwent extensive workup that failed to identify an underlying cause. In this sense, cannabis-induced acute pancreatitis may contribute to a small fraction of cases diagnosed with idiopathic acute pancreatitis. We add to the literature another case of cannabis-induced acute pancreatitis.

## Case presentation

A 48-year-old female patient presented with epigastric pain for the last three days associated with nausea and vomiting. Her epigastric pain was sharp in character and radiated to her back. It was not relieved with anti-acids and was not related to food. She denied fever, chills, weight changes, diarrhea, constipation, jaundice, hematemesis, hematochezia, melena, chest pain, shortness of breath, or cough. She had a history of type 2 diabetes mellitus, essential hypertension, hyperlipidemia, and gastroesophageal reflux disease (GERD). She had no previous history of pancreatitis, peptic ulcer disease, or coronary artery disease. She underwent cholecystectomy four years ago. Her family history was insignificant. Social history was negative for tobacco or alcohol use, but the patient admitted to daily heavy marijuana use for the last three years. Home medications included metformin and glipizide for diabetes, lisinopril, and amlodipine for hypertension, simvastatin for hyperlipidemia, and omeprazole for GERD.

On physical exam, the patient was in severe pain but was alert and oriented. Vital signs demonstrated a temperature of 36.5° C, blood pressure of 136/101 mmHg, heart rate of 87 beats per minute, respiratory rate of 18 breaths per minute, and O2 saturation of 99% on room air. Cardiovascular and lung exams were unremarkable. Abdominal exam was significant for moderate tenderness in the epigastric area and hypoactive bowel sounds without masses or rigidity.

Initial laboratory work showed a normal complete blood count (CBC) and basic metabolic panel (BMP) except for elevated glucose of 270 mg/dL. She had mildly elevated aspartate aminotransferase (AST) of 53 U/L and alanine aminotransferase (ALT) of 47 U/L. Her alkaline phosphatase, total bilirubin, and albumin were within normal limits. Serial troponin I levels were negative. Her lipase was elevated at 725 U/L. Triglyceride level was 156 mg/dL and serum calcium level was 9.7 mg/dL. CT abdomen and pelvis with contrast did not show acute abnormalities within the pancreas.

**Figure 1:**
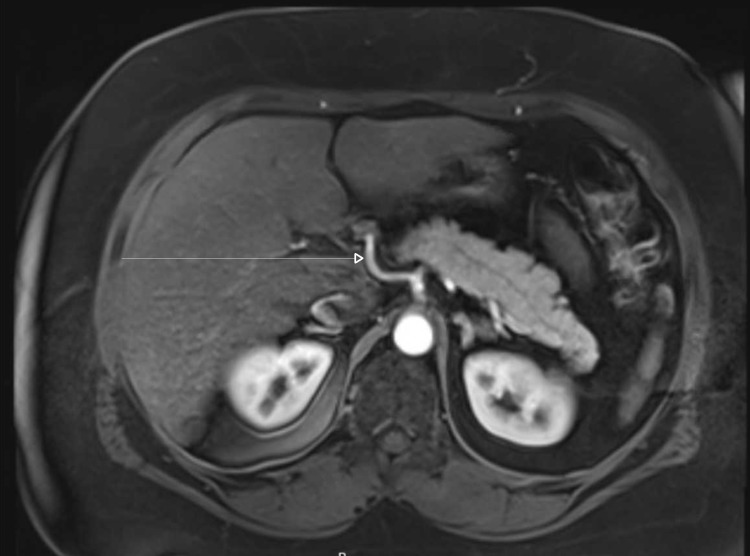
Magnetic resonance cholangiopancreatography shows no choledocholithiasis, or pancreatic mass to correlate with the patient's pancreatitis.

A diagnosis of acute pancreatitis was made, but the etiology of the patient’s pancreatitis was not clearly apparent. Initial ultrasound of the abdomen showed an absent gallbladder and no biliary duct abnormalities. Magnetic resonance cholangiopancreatography (MRCP) showed enlargement of the pancreatic duct but no choledocholithiasis (Figure 1).

The patient was treated with bowel rest, intravenous fluids, and morphine for pain control. Her abdominal pain improved after a couple of days and she was able to tolerate a regular diet. She had no complications during her hospitalization and was discharged the following day. At the time of discharge, she was counseled against marijuana use and was advised to quit. She was also informed that the cause of her acute pancreatitis was unclear, but the most likely culprit was her heavy marijuana use.

## Discussion

Cannabis is the most common psychoactive substance used worldwide. Relative to other psychoactive substances, cannabis is responsible for a small proportion of the health problems caused by substance use [7]. Long-term use of cannabis is associated with schizophrenia when used in adolescence in addition to alteration of brain structure and function [8-9]. Short-term use can lead to fatal motor vehicle accidents, acute psychosis, anxiety, hyperemesis, and airway irritation [10-14].

Cannabis-induced acute pancreatitis is a rare cause of acute pancreatitis. The diagnosis of cannabis-induced acute pancreatitis is challenging because clear diagnostic criteria are lacking. The diagnosis was made in the literature by a history of marijuana use in patients where other causes of acute pancreatitis were excluded. Causes frequently screened for included gallstones, alcohol, hypertriglyceridemia, hypercalcemia, post-ERCP, medications, and infections [15].

The pathophysiology of cannabis-induced acute pancreatitis is poorly understood. Cannabis exerts its action on the body through two receptors: CB1 and CB2. Both receptors are found in the pancreas and their activation may affect the pancreatic tissue [16]. However, the exact mechanism by which activation of these receptors can lead to acute pancreatitis remains unknown.

Cases reported in the literature usually had a history of heavy and daily use of marijuana. Most of them stopped using marijuana after their pain started and had a resolution of their pancreatitis after the withdrawal of marijuana. The clinical course was usually benign, and most patients had no recurrence after the first episode [4].

Our patient presented with an episode of acute pancreatitis where the cause was not immediately obvious. She had a history of cholecystectomy and her biliary tree on MRCP was unremarkable. She had no history of alcohol use and denied recent changes in her medications. Her serum triglycerides and calcium were within normal limits. The only significant component of her history was her daily heavy marijuana use. The diagnosis of cannabis-induced acute pancreatitis was made in our patient after other causes were excluded.

## Conclusions

Although the role of cannabis in acute pancreatitis is not fully understood, cannabis use should be considered in the differential diagnosis of idiopathic acute pancreatitis. In cases where initial evaluation fails to reveal a cause, it might be worthwhile to screen for cannabis use with careful history and/or urine toxicology screen. Further research is recommended to understand the pathophysiology behind cannabis-induced acute pancreatitis.
